# Why should stakeholders consider the effect of tensions in collaborative innovation in healthcare—lessons learned from surveying integrated care projects in Germany

**DOI:** 10.1186/s12913-023-10323-y

**Published:** 2023-11-23

**Authors:** Malte Haring, Juliane Schiller, Martin Gersch, Volker Amelung

**Affiliations:** 1 Hannover Medical School, Institute of Epidemiology, Social Medicine and Health System Research, Carl-Neuberg-Str. 1, Hannover, 30625 Germany; 2 inav– Private Institute for Applied Health Services Research GmbH, Schiffbauerdamm 12, Berlin, 10117 Germany; 3 Department of Information Systems, Freie Universität Berlin, Garystr. 21, Berlin, 14195 Germany

**Keywords:** Integrated care projects, Innovation, Implementation, Contradictions, Tensions

## Abstract

**Introduction:**

The German Innovation Fund supports projects that aim to improve healthcare through integration and intersectoral collaboration. As is typical for collaborative innovation projects, partners often pursue different objectives, which can create tensions and affect outcomes. The study aims to explore the causes and effects of tensions in integrated care projects and how frameworks, processes, and management should be designed to deal with tensions and achieve their productive effects.

**Methods:**

In an online survey we asked participants about the causes, effects, and management of tensions and their implications for integrated care projects (*n* = 58 completed questionnaires). We applied bivariate descriptive statistics to analyse the quantitative data.

**Results:**

Tensions between stakeholders, caused by deep-seated differences and the design of the project frameworks, often affect the course and outcome of innovative integrated care projects. However, through appropriate conflict management and negotiation processes such tensions can be managed constructively and lead to better outcomes.

**Discussion:**

Tension is usually seen as something unpleasant to be avoided and/or overcome. In fact, tensions can have positive effects, the importance of which remains little understood. Developing appropriate frameworks for managing and integrating different perspectives are key factors in unlocking the positive potential of tensions in integrated care projects.

**Supplementary Information:**

The online version contains supplementary material available at 10.1186/s12913-023-10323-y.

## Background

### Introduction

Integrated care holds out the promise of improved quality of care for patients. However, the patient journeys resulting from the requirements for integration and the reduction of barriers between the actors involved are characterized by a high degree of complexity. Therefore, like other innovations in the health care system, the implementation of integrated care is characterized by difficulties and barriers in the cooperation of the actors [1, 2]. This is particularly true for the German healthcare system, which, although it has the second highest spending in the world in relation to GDP, and provides a wide range of services and resources, only performs at an average level compared to other industrialized countries [3]. The main causes of this underperformance are the economic and competitive framework conditions, lack of coordination, strong historic sectoral separation and institutionalisation, which characterise the German system and are also responsible for the sluggish implementation of integrated care [4–6]. To improve this situation and promote integrated care innovations in the German health system, the Innovation Fund (IF) was established. The aim of the €2 billion fund is to provide support for innovative projects that pursue the implementation and research of innovative and collaborative, cross-sectoral concepts for the further development of existing care structures [7–9].

**Fig. 1:**
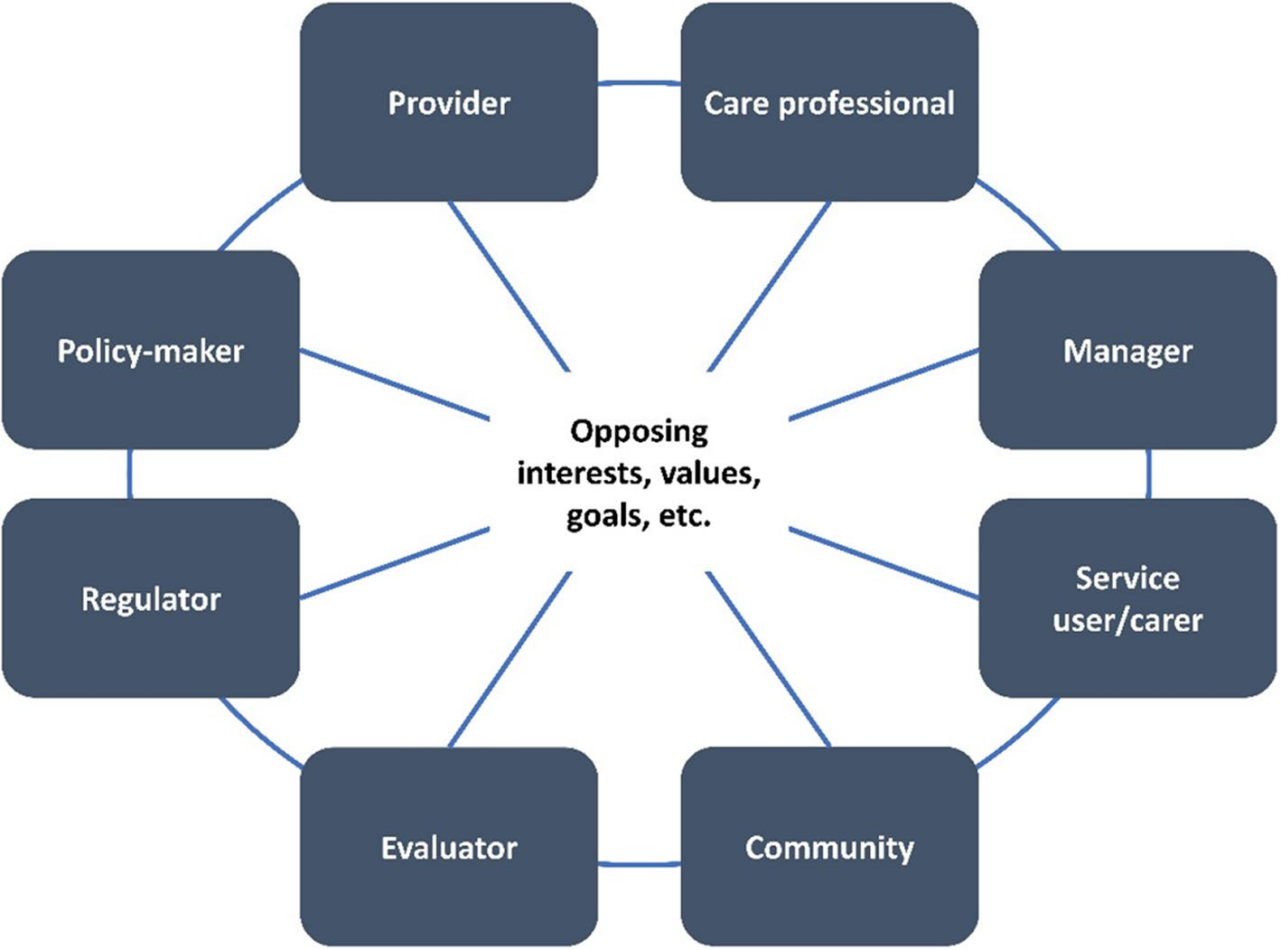
Different stakeholder groups that are involved in IF consortia, based on Shaw et al., 2011 [17]

Approved IF projects are formed as time-limited, cross-sectoral project consortia consisting of a consortium leader and participating partners from statutory health insurances, service providers, scientific research institutions and other organisations. Consortia follow a common consortium agreement and budget structure, which correspond to the concept of network relationships as defined by Oliver Williamson, and other theoretical concepts on networks, including healthcare networks [10–13]. Cross-sectoral cooperation and collaboration within established institutional arrangements is one of the central features of the IF [7]. It is well-established then, that consortia stakeholders have different expectations and objectives, shaped by their institutional affiliation, cultures, and identities. The integration of conflicting interests, values and expectations of project members increases the complexity and coordination efforts involved in managing internal project relationships and thus represents one of the central challenges of not only consortia but of integrated care itself (see Fig. 1) [14, 15]. The different perspectives also increase the demands on the management and governance of projects. It is therefore necessary to respond to the changing situations and to deal constructively with the interests of project partners [16].

The challenge posed by these consortia, then, is to manage, long-term, a multitude of inherent tensions between the consortium partners arising from fundamental, deep-seated differences (interprofessional, intersectoral, interdisciplinary, and interorganisational) and to aim to integrate opposing poles. For example, the pursuit of autonomy often conflicts with the recognition of (inter-)dependencies between partners aiming for integrated care, while trust in partners may be hampered by the need for control [1, 18–23].

**Fig. 2:**
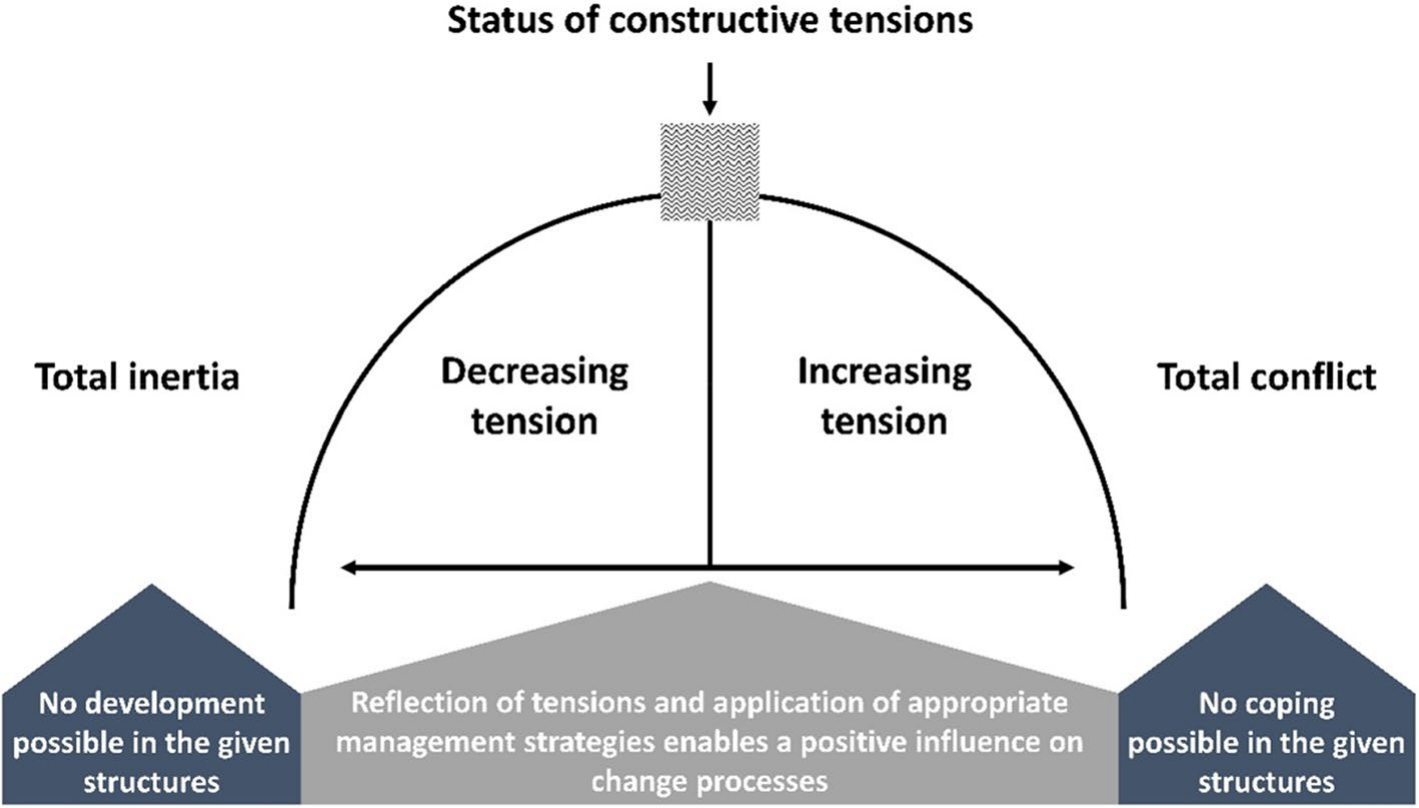
Finding the right balance of tension, adapted from Hurrelmann & Richter, 2013, p. 125 [24]

Building on existing research [22, 23], we assume that tensions inherent in integrated care can have both advantages (e.g., promoting debate and leading to open discussions and potential agreement on a common goal) and disadvantages (e.g., leading to an open conflict that remains unresolved and could prevent the innovation process coming to fruition) and hypothesize that there is a balance between tensions being constructive rather than destructive (see Fig. 2).

To validate this assumption, we collect and compare experiences from across projects supported by the IF. The IF provides a standardized framework of the process that such collaborative healthcare innovation projects undergo, and therefore offers an ideal real-world scenario to observe how tensions play out in integrated care, multi-partner, and multi-year innovation projects.

### Objectives of the innovation fund

The decision to establish a fund in Germany for healthcare innovations resulted from the experiences gained in the context of initiatives that have promoted integrated care since the turn of the millennium [7]. The initiatives’ financial and competitive framework conditions were deemed unsatisfactory in terms of promoting sustainable innovation because of, on the one hand, increased requirements for economic efficiency and, on the other, lack of clarity about the responsibilities of the stakeholders involved in care and remuneration mechanisms [25]. In response, a new public fund was set up to develop integrated care structures in Germany by providing targeted support for innovation projects improving intersectoral care and with the sufficient potential to be permanently embedded in the healthcare system. In addition, the fund aimed at providing both a monetary and a non-material incentive to overcoming these barriers [9, 26].

The Innovation Fund was established in 2015, extended in 2019 and made permanent in 2021 [8, 27, 28] to promote innovation and integrated care in the health system. Its budget allocation was €300 million per year between 2016 and 2019, and €200 million annually from 2020 to 2024, resulting in a total of €2 billion euros over the entire funding period. The fund will finance projects that a) pilot new forms of care that go beyond the current standard of care (‘Neue Versorgungsformen’, or NVF) and that b) focus on health services research to that effect (‘Versorgungsforschung’, or VF). To date, a total of 535 projects have been approved for funding by the IF [26].

**Fig. 3:**
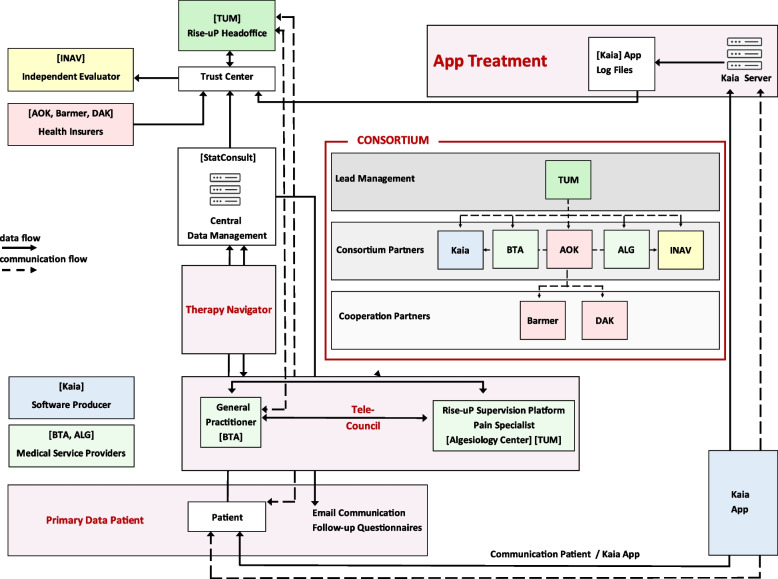
Collaboration between different stakeholders in the innovation fund project *Rise-uP* based on Priebe et al., 2022 [30]

For the present paper, we focus on NVF, and with an IF project we refer to projects in this category. These projects directly address the topic of integrated care and cooperation between sectors, care areas and professional groups. Examples of NVF projects include *TELnet@NRW*, a cross-sectoral telemedicine network for infectious diseases, which links general practitioners, hospital physicians and non-medical professional groups, and *Rise-uP*, which involves medical service providers, health insurers, research institutes, and other partners such as software producers (see Fig. 3) [29, 30].

### Working structure and functionality of the innovation fund

**Fig. 4:**
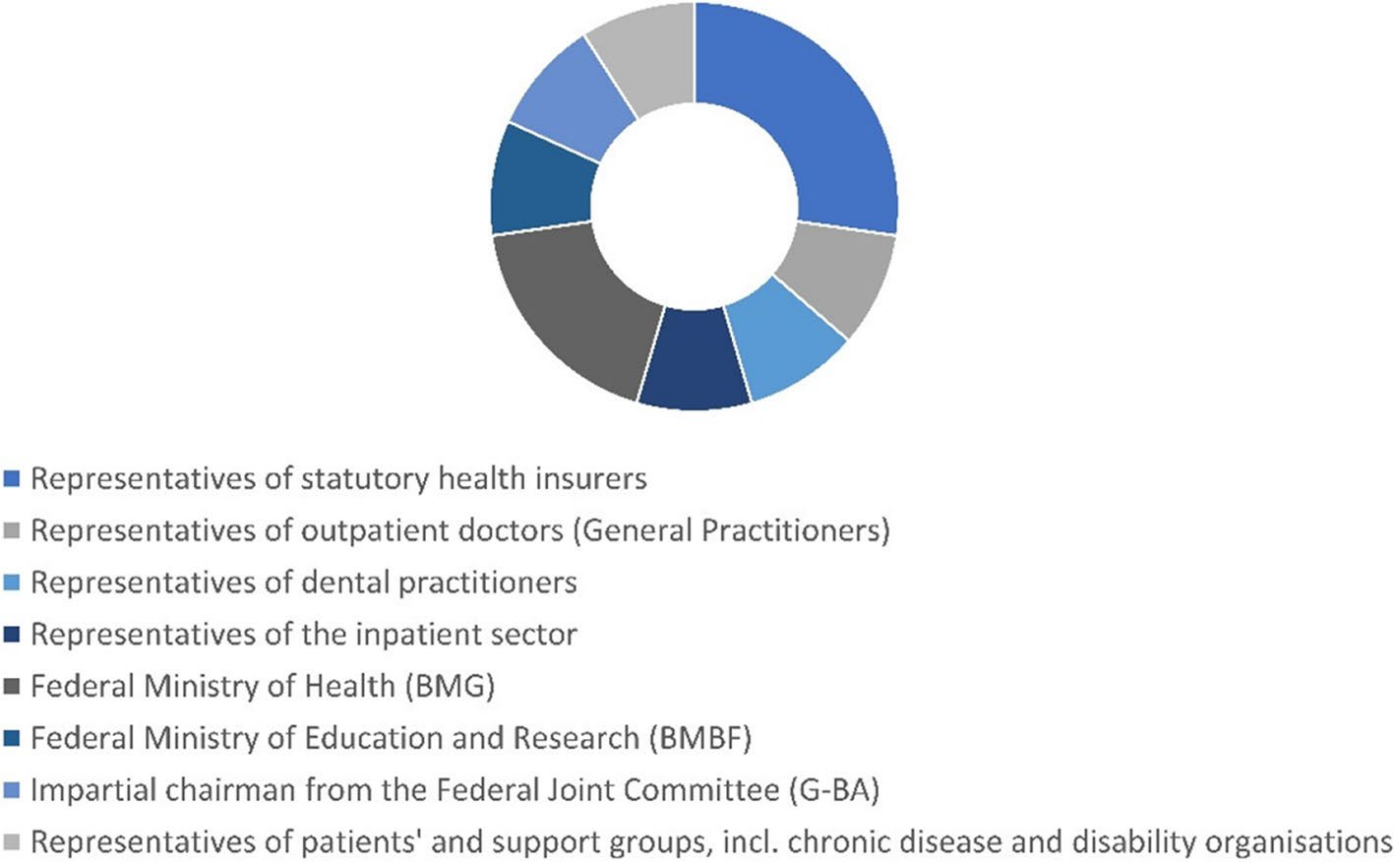
Composition of the innovation committee based on G-BA, 2022 [26]

The purpose of the IF was defined in response to a call for cross-sectoral cooperation and the structural integration of different sectors and stakeholders of the health system. The traditional stakeholders of the German health care system were involved in the design and implementation of the fund as can be seen, for example, in the composition of the Innovation Committee, which sets the priorities and criteria for the allocation of financial resources from the fund and assesses the applications it receives (see Fig. 4) [31, 32].

**Fig. 5:**
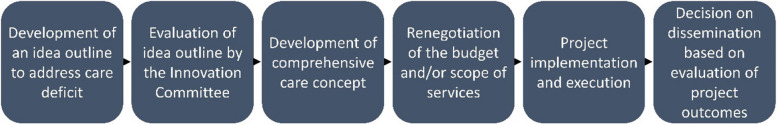
Application process in the IF based on G-BA, 2022 and Deh, 2021 [34, 35]

Applicants to the IF must demonstrate that they meet the described funding criteria and other requirements. The procedure of the process can be seen in Fig. 5 and corresponds to findings from innovation research regarding the life cycle of innovations [33].

IF projects can be implemented either by one applicant or by several partners forming a consortium. In the latter case, partners sign a consortium contract and a funding agreement, which sets out the joint budget and its allocation between partners. Project consortia consist of a consortium leader and consortium partners [34].

### Working across sectors in the network

In order for novel forms of care delivery to develop between consortia partners who each hold their own views and expectations on how care services ought to be provided – in line with their institutional affiliation, organisational objectives, perspectives, and interests [15, 16] – the IF framework explicitly encourages cross-sector collaboration. This, however, often presents considerable challenges to individual consortia members, apart from increasing the complexity of managing each IF consortium. The kind of challenges faced by project members are well known from integrated care networks in general and can be described as a) interorganisational, b) intersectoral, c) interprofessional, and d) interdisciplinary. In this context, for such diverse partners to collaborate successfully and develop new relationships and constellations for the delivery of integrated care services, requires each to challenge their own organisation’s framework and boundaries while engaging with (and learning from) each other's perspectives. It is, however, the very plurality arising from their cooperation (and the prospect of change made to the processes of care implementation) that can give rise to conflicts between partners. The potential for tensions is further intensified by the inherent interdependency between consortium members, the shared financial budget, and the strictly regulated framework conditions of the IF. Such tensions between consortium partners can have a detrimental impact on the implementation and the outcomes of a project, even leading to failure [22, 23].

### Organisational tensions

**Fig. 6:**
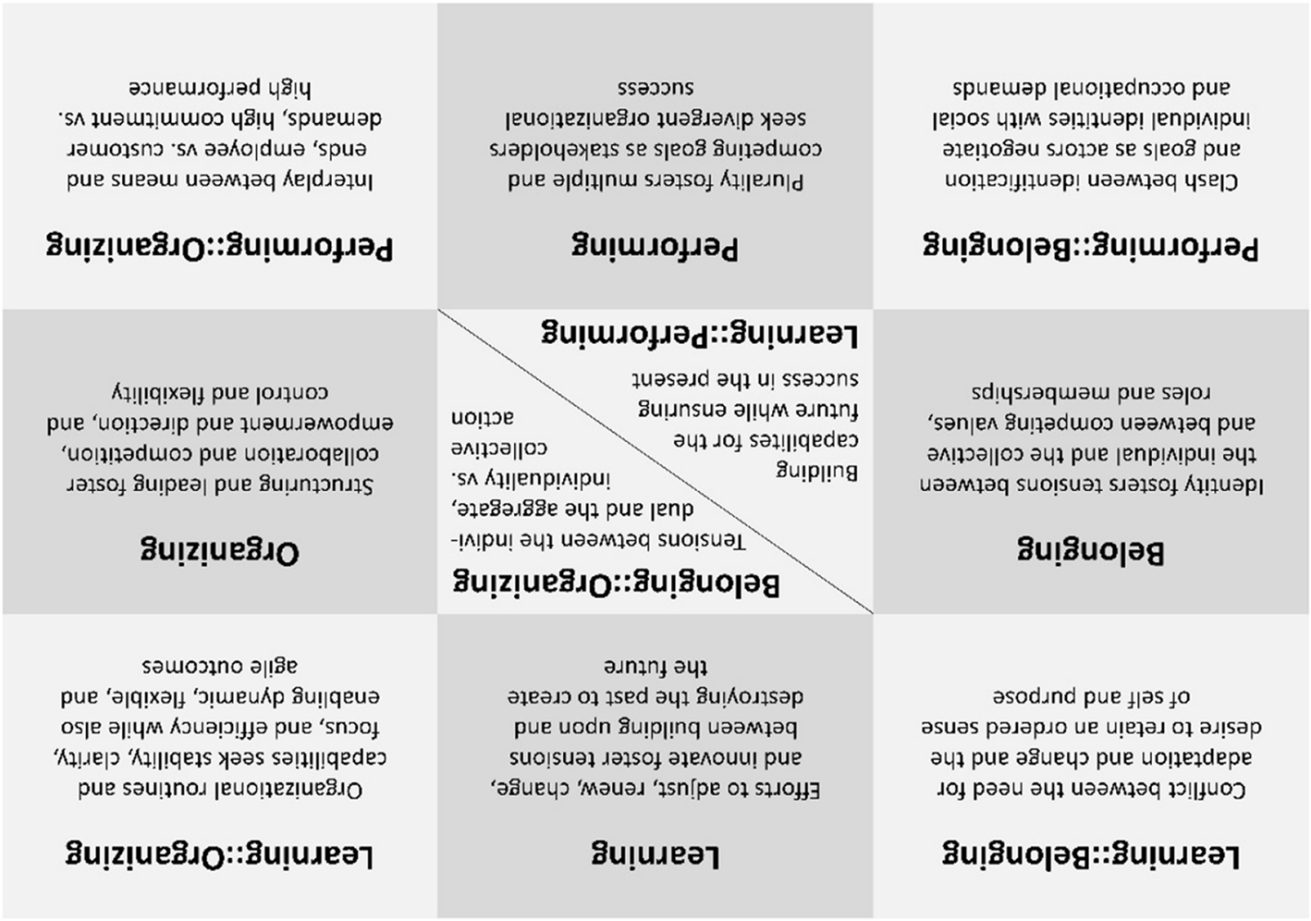
Categories of organisational tension according to Smith and Lewis, 2011 [23]

Tensions refer to conflicts that arise from opposing, sometimes contradictory views and requirements in change processes (i.e., the implementation of an innovation). They can range from simple discrepancies and differing views among consortium partners to more fundamental and even irreconcilable contradictions or paradoxes. When different beliefs and perspectives collide, tensions become apparent, which represent both the observable symptoms and the underlying causes of these conflicts [22, 23, 36]. Smith and Lewis have identified and described four main categories of organisational tensions that help to understand how such tensions arise and what is causing them. In addition, there are also six cross-cutting categories that can be used to classify tensions that do not fall neatly into one category but rather straddle two (see Fig. 6) [23].

This analysis and categorization of organisational tensions in change processes is important, since the occurrence of tensions attracts the attention of the actors involved and requires them to react to and engage with the issues. Tensions can thus trigger unexpected changes during the innovation process [22, 23, 37–39]. The consideration of tensions is well established in different organisational contexts including in the contexts of integrated care and innovations in healthcare systems [18–21]. In addition to analysing the causes and dimensions of tensions, managing these is also of central importance to the successful implementation of projects [22, 23, 36].

### Managing organisational tensions

**Fig. 7:**
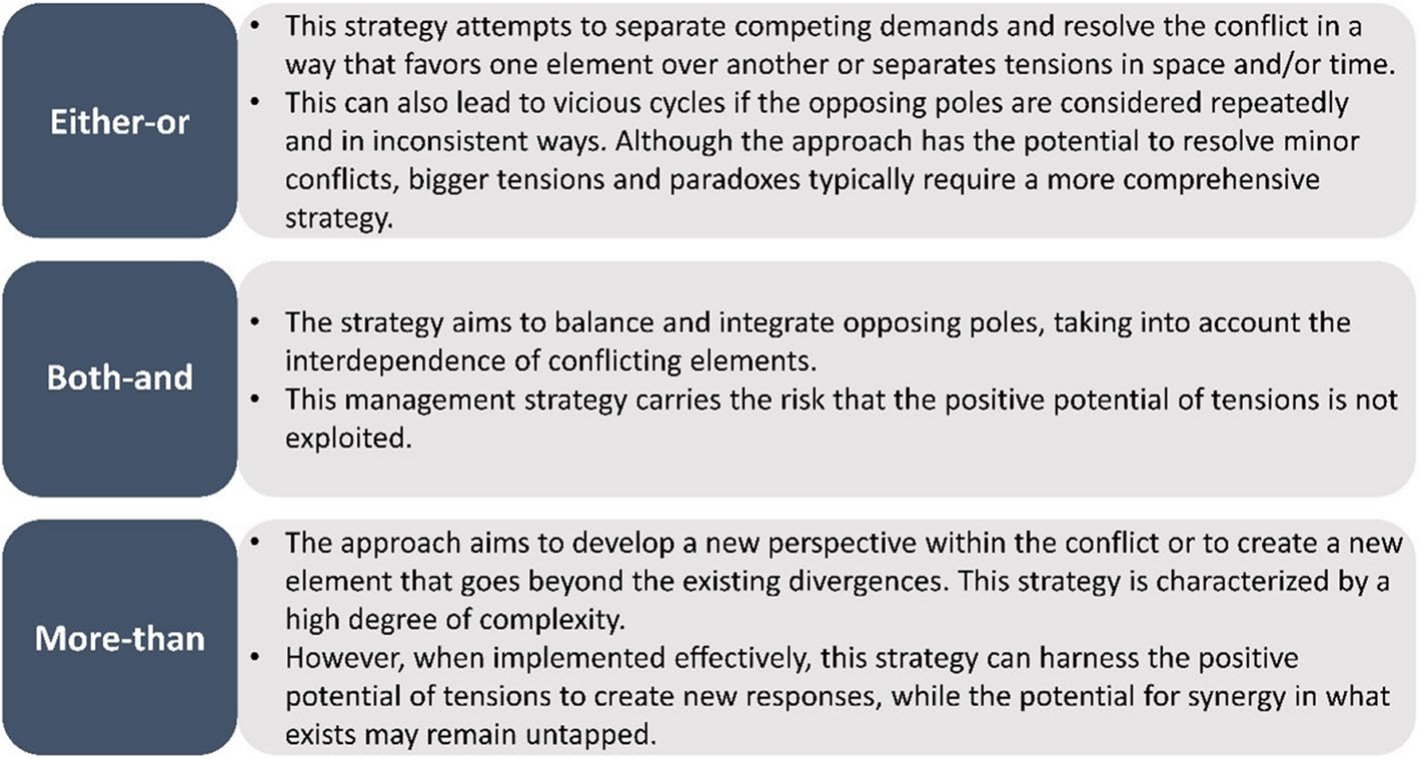
Three strategies to deal with tensions [22, 23, 36, 37, 40]

The application of suitable management strategies is decisive in influencing not only how well opposing views and tensions are dealt with but also how purposefully their activation potential can be used to bring about the desired changes [23]. The effective management of tensions and their associated dynamics should also consider the creative potential of tensions, while providing answers to dealing with divergent requirements. In this way, constructive effects can be achieved (virtuous cycles) and the exploitation of these creative potentials can lead to synergies that promote the change and development of an organisation. If, on the other hand, organisational tensions or conflicts are left simmering below the surface or attempts made to resolve them as quickly as possible, it could jeopardise the very development (let alone the successful implementation) of innovations (vicious cycles). A potential solution would be to deal with tensions and partner dynamics in a reflective and productive manner, which requires the selection of adequate approaches to respond appropriately to the different types and strengths of tensions [20, 22, 23, 37, 38]. The literature has suggested 3 management strategies to be essential for promoting virtuous cycles and avoiding vicious cycles (see Fig. 7).

### Tensions in the innovation fund

Among the actors that are typically brought together in an IF cooperation, some will be new to the experience of engaging in integrated cooperation and collaboration on an innovation. It is, however, precisely because actors pursue different interests and perspectives on the goals, values and methods of healthcare that integrated care concepts have been brought into existence [14]. Indeed, these contradictions are instrumental in shaping the innovation process pursued by IF projects and, hence, the repeated occurrence of tensions forms an integral – some may say inevitable – part of its trajectory. Even the clash of values and organisational cultures of the stakeholders involved can be seen as a key factor in shaping the development of the health system beyond the rigid status quo [14, 36, 39, 41].

The example of the standardized innovation process in IF projects can therefore be used to investigate whether such cross-sector collaborations, where stakeholders are virtually forced out of their comfort zones, can pave the way to successful integration, spark constructive debates between the actors and thus lead to better results (i.e., the intended outcome of improving integrated care), or whether, on the contrary, the resulting tensions outweigh the benefits of the projects and may cause lasting damage. Thus, IF projects provide an arena for the systematic collection and comparison of experiences from different integrated care innovation projects and from stakeholder collaboration in achieving care innovation. This is necessary to understand the causes, effects, and systematics of tensions at the micro, meso, or macro level and their impact on integrated care and the implementation of care innovations in project consortia working together across sectors [22, 23].

Therefore, the aim of this study was to a) explore the nature of tensions in IF projects and their causes, b) assess how these tensions affect projects and, in particular, their outcomes, and c) examine how the framework conditions and the implementation, administration and management of integrated care projects need to be designed to deal with tensions and achieve their productive effects.

This knowledge will contribute not only to improving integrated care, but also to enhancing the effectiveness of the Innovation Fund’s support, taking into account the unavoidable occurrence of tensions between diverse stakeholders.

## Research methods

### Data collection

The survey, which is available as a supplementary file to this article, combined questions with single and multiple choices as well as open questions and was conducted using the LimeSurvey survey tool between June and October 2022. The survey involved stakeholders from project consortia, i.e., stakeholders from health insurers, service providers, scientific research institutions, and other organisations. The 20 items of the questionnaire were constructed to collect data on the causes, effects, and management of tensions, and on the implications for integrated care using the example of the IF projects. All participants had to answer the same questions, i.e., no adaptive questioning was applied.

The survey instrument was developed by the authors of this study in a multi-stage process. First, a basic structure of the survey was developed based on the findings of a systematic literature review [19]. In this context, the types, causes, handling and effects of tensions in particular were identified as important topics to be pursued further and mapped accordingly as dimensions in the questionnaire. The questions and response options developed and derived from this were validated in six preparatory expert discussions, in which relevant stakeholder groups with a range of roles in IF projects were represented. The survey instrument was pretested involving experts from IF projects.

To test our hypotheses, ten of the 18 quantitative questions in the survey (nos. 1–17, 20), were formulated as statements with which respondents could indicate their agreement or disagreement on a 5-point Likert scale. For the remaining questions, we deliberately deviated from this scheme by including multiple answer options in seven questions and an additional free-field response field when “Other” was ticked, to allow for a wider range of answer options in a nevertheless closed question design. We were thus able to collect qualitative data through the two open questions in which participants could express themselves (nos. 18, 19). Apart from these questions, the survey questions were programmed as mandatory fields that could not be skipped in the online survey (see Table 1). The open-ended questions are cited and discussed in the results section. It should be noted that the two open-ended questions were not the focus of our analysis. Rather, in the spirit of qualitative research, it was an in-depth look to provide context and detailed explanations of participants' personal experiences of the negative or positive experiences of tension and conflict in IF projects. The goal in asking the two questions was to gather detailed responses—as compared to survey responses—to two selected questions that provide important insights for our analysis.

**Table 1:** Presentation of response frequencies

Variable	n (sample size = 58)	% (Cases)	% (Participants)
**What is your institutional affiliation? [Survey question 20]**			
University/scientific institute	30	52%	
Health insurance	14	24%	
Health care provider	7	12%	
Other	6	10%	
Manufacturer of a digital application/platform	1	2%	
**The combination of different institutional identities and logics in the consortium contains considerable potential for tension. (median = 4: Agree) [Survey question 3]**			
Strongly agree	11	19%	
Agree	20	34%	
Neither agree nor disagree	16	28%	
Disagree	10	17%	
Strongly disagree	1	2%	
**The structural framework of Innovation Fund projects contains considerable potential for tension. (median = 4: Agree) [Survey question 5]**			
Strongly agree	16	28%	
Agree	18	31%	
Neither agree nor disagree	19	33%	
Disagree	4	7%	
Strongly disagree	1	2%	
**What fundamental differences occurred during the course of the Innovation Fund project? (Multiple answers possible) [Survey question 1]**			
Consortium partners have different working methods	45	36%	78%
Consortium partners have different ideas about cooperation	33	27%	57%
Consortium partners work to different time horizons	22	18%	38%
Consortium partners pursue different objectives	16	13%	28%
Other	8	6%	14%
**In which phase of the Innovation Fund project did fundamental differences between the consortium partners (individuals/interest groups) become apparent? [Survey question 6]**			
Project Implementation Phase (in terms of content)	44	35%	76%
Project Implementation Phase (administrative aspects)	32	26%	55%
Project Development and Application Phase	15	12%	26%
Project Completion Phase	13	10%	22%
Project Transfer Phase	8	6%	14%
Solution Finding Phase	8	6%	14%
Problem Identification Phase	5	4%	9%
**Which personal circumstances reinforce the fundamental differences between the consortium partners? (Multiple answers possible) [Survey question 2]**			
Characteristics of the individual parties involved	33	32%	57%
Lack of understanding of the opinions and characteristics of the other consortium partners	27	26%	47%
Lack of transparency and mutual trust between consortium partners	19	19%	33%
Existing cooperation experiences between the institutions of the consortium partners	12	12%	21%
Other	11	11%	19%
**Which structural factors reinforce the fundamental differences between the consortium partners? [Survey question 4]**			
General framework and funding structure (e.g., no room for budgetary or content adjustments, tendering procedure)	38	28%	66%
Complexity of project execution in terms of content (e.g., difficulties in recruitment or influence of other actors)	37	27%	64%
Bureaucratic requirements (e.g., reporting and documentation requirements)	32	23%	55%
Contractual arrangements for project execution (e.g., performance-based dependency, lack of binding force)	27	20%	47%
Other	4	3%	7%
**How did the fundamental differences affect the course of the Innovation Fund project? [Survey question 7]**			
Strongly divergent views regarding the willingness to change established procedures and working methods (e.g., existing processes in collaboration vs. new processes in the project)	24	29%	41%
Strongly divergent views regarding the interests to be pursued (e.g., competing values, competing understanding of roles)	18	22%	31%
Strongly divergent views regarding structures and processes in the project (e.g., control vs. flexibility, collaboration vs. competition)	15	18%	26%
Strongly divergent views regarding project objectives (e.g., efficiency vs. quality, stability vs. dynamics)	13	16%	22%
Other	12	15%	21%
**What are the institutional affiliations of the consortium partners (individuals/stakeholders) where these impacts have become apparent? (Multiple answers possible) [Survey question 8]**			
University/scientific institute	29	24%	50%
Health care provider	26	22%	45%
Health insurance	24	20%	41%
Manufacturer of a digital application/platform	17	14%	29%
Association of Statutory Health Insurance Physicians/Professional Association	15	13%	26%
Other	8	7%	14%
**The effects of fundamental differences are usually predictable, and resulting conflicts can be avoided by planning well in advance. (median = 3: Neither agree nor disagree) [Survey question 9]**			
Strongly agree	5	9%	
Agree	16	28%	
Neither agree nor disagree	18	31%	
Disagree	16	28%	
Strongly disagree	3	5%	
**The effects of fundamental differences cannot be avoided in general and require a dynamic approach to dealing with the resulting tensions or conflicts. (median = 4: Agree) [Survey question 10]**			
Strongly agree	18	31%	
Agree	24	41%	
Neither agree nor disagree	11	19%	
Disagree	5	9%	
**STRONGLY DISAGREE**	0	0%	
**The occurrence of tensions and conflicts as a result of fundamental differences influences the progress of Innovation Fund projects. (median = 4: Agree) [Survey question 11]**			
Strongly agree	14	24%	
Agree	24	41%	
Neither agree nor disagree	18	31%	
Disagree	1	2%	
Strongly disagree	1	2%	
**The occurrence of tensions and conflicts as a result of fundamental differences threatens the success of a project and poses an existential threat to it. (median = 3: Neither agree nor disagree) [Survey question 12]**			
Strongly agree	9	16%	
Agree	17	29%	
Neither agree nor disagree	20	34%	
Disagree	9	16%	
Strongly disagree	3	5%	
**Which approach to managing fundamental differences in the project helps to resolve any resulting conflicts? [Survey question 13]**			
Activities to provide the facilitation of consortium partners and their actions (e.g., regular project meetings for information, coordination, and transparency)	48	31%	83%
Activities to develop an understanding of the consortium partners (e.g., their goals, interests, and ways of working)	37	24%	64%
Activities to develop a mutual objective in the project (e.g., common milestones, discussion of requirements, deliverables, and procedures)	36	23%	62%
Activities to reduce the requirements associated with the funding structure and processes (e.g., facilitating and making financial and content adjustments more flexible)	30	19%	52%
Other	3	2%	5%
**Dealing with tensions and conflicts caused by fundamental differences has a negative impact on the course of the project (e.g., delays, deterioration of results, deterioration of the general atmosphere in the project) (median = 4: Agree) [Survey question 14]**			
Strongly agree	15	26%	
Agree	15	26%	
Neither agree nor disagree	14	24%	
Disagree	13	22%	
Strongly disagree	1	2%	
**Dealing with tensions and conflicts as a result of fundamental differences has negative effects extending beyond the course of the project (e.g., damage to trust between stakeholders). (median = 3: Neither agree nor disagree) [Survey question 15]**			
Strongly agree	7	12%	
Agree	14	24%	
Neither agree nor disagree	18	31%	
Disagree	15	26%	
Strongly disagree	4	7%	
**Dealing with tensions and conflicts as a result of fundamental differences has positive effects on the course of the project (e.g., creating interfaces, accepting different points of view, increasing awareness). (median = 3: Neither agree nor disagree) [Survey question 16]**			
Strongly agree	6	10%	
Agree	18	31%	
Neither agree nor disagree	20	34%	
Disagree	12	21%	
Strongly disagree	2	3%	
**Addressing tensions and conflicts resulting from fundamental differences has positive effects extending beyond the course of the project (e.g., creating a healthy level of discussion between different actors). (median = 3: Neither agree nor disagree) [Survey question 17]**			
Strongly agree	5	9%	
Agree	14	24%	
Neither agree nor disagree	23	40%	
Disagree	12	21%	
Strongly disagree	4	7%	

For the survey, the project leaders of the 194 approved projects of the NVF who were listed as contacts for the IF project on the official pages of the G-BA were contacted by e-mail with a covering letter of the investigator, describing the aim of the study and a link to the online questionnaire. Participants were also invited to share the survey with the members of their respective consortium. In total, the survey was sent to 185 individuals. This number results from the fact that in 12 cases addressees were project leaders of two or more IF projects, so that several projects with one contact were requested. On the other hand, multiple mailings to one project were also possible if several contact addresses or project leaders were given for this project. As part of the survey, cookies were set but no IP checks or log file analysis were performed to avoid multiple participations. Participants were informed about data protection, length of time of the survey and data security and had to give their consent to the survey. To increase the response rate, reminders were sent at two points in time.

### Data analysis

We performed univariate and bivariate data analyses. In a first step, descriptive statistical analyses to investigate the experiences and opinions of IF project members regarding fundamental contradictions, their characteristics and management in the context of cross-sectoral projects were undertaken. Thus, frequencies and percentages of categorical variables were calculated. Furthermore, the median of ordinal variables, collected using a Likert scale (Strongly disagree = 1; Disagree = 2; Neither agree nor disagree = 3; Agree = 4; Strongly agree = 5;), were determined. For better comprehension and clarity, responses to the 5-point Likert scales were grouped into 'Agree', 'Disagree' and 'Neither agree nor disagree' in the results section. The frequencies of all five scale items can be found in Table 1. To assess the statistical significance of differences and correlations between the characteristics of specific survey items, cross-tabulations were generated, and a Pearson’s chi-squared test was performed. If expected cell frequencies were below 5, Fisher’s exact test was applied. A correlation analysis was carried out exclusively for survey items that were expected to yield knowledge in the sense of the research question (cf. results section for further details). To deal with missing data, a complete case approach was applied. No statistical correction to adjust for the non-representative sample was applied. All quantitative analyses were conducted using R Studio version 2022.07.1 and Microsoft Excel Version 2211.

While the answers to the two open-ended questions were evaluated in two different ways, each was based on a content analysis of the qualitative data, using Microsoft Excel Version 2211 for data management.

The responses to the open-ended questions were qualitatively synthesized using a thematic analysis approach [42] and following a deductive (no. 18) or inductive (no. 19) logic, applying a classified theory-based classification into the tension categories according to Smith and Lewis [23] for question no. 18.

## Results

**Fig. 8:**
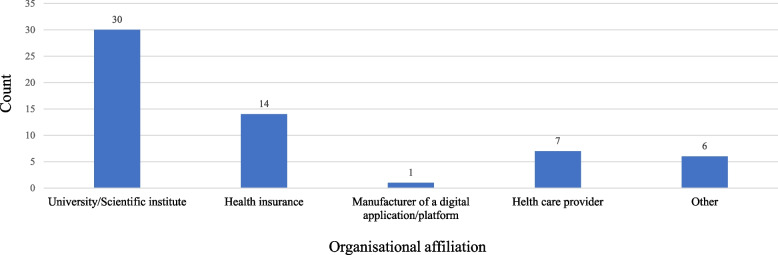
Frequency of participants’ institutional/organisational affiliations

A total of 83 project partners participated in the survey. The survey was sent out by project members to 185 people, all of them being project leaders in the 194 approved projects of the NVF, which corresponds to a response rate of 45%. However, since the survey could be forwarded by the addressees, i.e. ‘open survey’, the validity of the response rate is limited. We only included completed questionnaires, i.e., responses from 58 participants, in the analysis (completion rate = 31%). Of these, 52% (*n* = 30) of these were affiliated with a university or research institution, 24% (*n* = 14) with a health insurance, 12% (*n* = 7) were health care providers, and one (2%) was a manufacturer of a digital application or platform. Ten percent (*n* = 6) of participants had other institutional affiliations, e.g., a private service provider [*n* = 1], health office [*n* = 1], foundation [*n* = 1], health network [*n* = 1], project management offices [*n* = 2] (see Fig. 8).

More than half of the participants found that the source for potential tensions was inherent in the combination of different institutional identities and logics in the consortium (53%, *n* = 31), and the structural framework itself (59%, *n* = 34).

The participants were asked what fundamental differences occurred during the IF project, in which phase of the project these became apparent, and which personal circumstances and structural factors reinforced them. Regarding fundamental differences, different working methods (78%, *n* = 45) and different ideas about the cooperation of the consortium partners (57%, *n* = 33) were the most frequently selected differences. According to the majority of participants, fundamental differences became apparent during the implementation phase of the project. This refers to the implementation of the project in terms of content (76%, *n* = 44), e.g., the realisation of work packages and adjustment to unforeseen events and to the project administration (55%, *n* = 32), e.g., the reporting, communication and operational project management. These differences were mostly reinforced by the characteristics of the individual parties involved (57%, *n* = 33) and by failing to understand these characteristics and differences in opinions (47%, *n* = 27).

The main structural factors that the survey identified as magnifying the differences between the consortium partners were, respectively, the general framework and funding structure (66%, *n* = 38), the sheer complexity of the projects (64%, *n* = 37), and bureaucratic requirements (55%, *n* = 32). When asked how the fundamental differences in fact influenced the course of the IF project, strongly divergent views regarding the willingness to change established procedure and working methods seemed to be a central factor (41%, *n* = 24). Universities and scientific institutes (50%, *n* = 29), health care providers (45%, *n* = 26) and health insurances (41%, *n* = 24) were the institutional affiliations being impacted the most by the fundamental differences.

Regarding the predictability of the differences between partners and the avoidability of their effects through good planning, no clear statement can be made on the basis of the survey results (Agree 37%, *n* = 21; Disagree 33%, *n* = 19; Neither agree nor disagree 31%, *n* = 18). Nonetheless, 71% of participants agreed on the importance of applying a dynamic approach to dealing with tensions that arise because of fundamental differences between consortium partners.

**Fig. 9:**
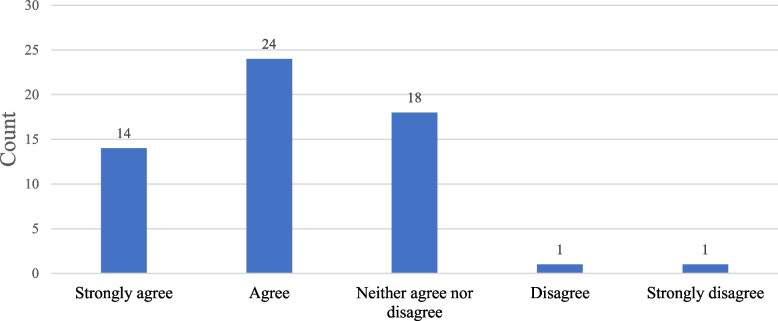
Impact of tensions on the progress of IF projects as perceived by survey participants

As for the actual impact of tensions on the progress of IF projects, the picture also became clearer (cf. Fig. 9). Almost two-thirds of participants (65%, *n* = 38) agreed that tensions and conflicts resulting from fundamental differences impede the progress of IF projects, and nearly half (45%, *n* = 26) thought that these tensions can threaten a project's success.

Our research went one step further and asked participants about approaches that they thought could help manage tensions in IF projects. The activities that participants considered to be potentially the most helpful were, respectively, facilitation of consortium partners and their actions (83%, *n* = 48) (e.g. regular meetings), the development of a shared understanding of the goals and working methods of consortium partners (64%, *n* = 37) and of shared project objectives, (62%, *n* = 36). Facilitating communication and joint planning within IF projects therefore also seem essential to managing tensions in a beneficial way.

The need to deal with tensions and conflicts within IF projects was perceived as negatively impacting project performance (e.g. by delays) by over half of the participants (52%, *n* = 30). Asked whether the tensions had a negative impact on the project beyond its lifespan, the results are less clear: 36% (*n* = 21) agreed, 33% (*n* = 19) disagreed, and 31% (*n* = 18) of participants took a neutral position. However, the survey results also show that dealing with tensions was seen as positive, with 41% of participants agreeing that dealing with tensions can have positive effects on the project*.* As with negative effects, the positive impact of tensions beyond the project is also inconclusive, according to the survey results, with 33% (*n* = 19) of participants agreeing, 28% (*n* = 16) disagreeing, and most taking a neutral position (40%, *n* = 23).

Part of our research was to better understand whether actors' organisational affiliations impacted on their perception of the fundamental differences that occur in an IF project. A two-sided Fisher’s exact test revealed no statistically significant association between these categorical variables (*p* = 0.98). Further correlation analyses that were expected to yield knowledge in the sense of the research question were performed but did not reveal any significant associations. These analyses are available upon request.

The survey also included two open-ended questions. First, participants were asked about their most negative experiences with tensions and conflicts in an IF project and what, in hindsight, they identified as their source. 45 participants responded to this question, describing 40 types of tensions. 80% of the reported tensions could be classified into the categories *Performing, Belonging,* or *Performing::Belonging* according to Smith and Lewis [23]. Most types of tensions (72.5%, *n* = 29) were assigned to the *Performing* category (major) with more than half of these (58%, *n* = 17) being also somewhat linked to the *Belonging* tensions category. These types of tensions include conflicts between competing values and roles of stakeholders (Belonging) or individual and shared project goals (Performing), which are likely to occur in integrated care collaboration and are underscored by the following participant statements:

*[There are] tensions between business partners, who have clear medium-term objectives linked to revenue, and scientific or public institutes, who want to achieve long-term results in which revenue plays less of a role. Delays in the project are perceived differently by these different parties.*

*Different ways of working, friction between medical professionals (clinicians) and non-medical professionals regarding prioritization.*

*To improve recruitment, actions were needed that some health insurers did not want to go along with. Two health insurers therefore threatened to leave the project, which would have had a considerable impact on the entire course of the project. The reason for the health insurers was their organisational structures and internal guidelines, but also their pessimistic thinking. They lost sight of the common goal.*

Eighty-five percent of all tensions were caused by internal project conditions. Tensions caused by external conditions (15%, *n* = 6) were previously assigned to the category ‘Organizing’ in all cases.

The second open-ended question asked participants about their positive experiences with tensions and conflicts in IF projects. A total of 38 participants responded and, of those, 31 reported experiences of tensions, which we described and analysed. Text passages were classified regarding the type of activity performed (‘Communication and discussion’, ‘Joint target development’, ‘Coordination’), the result (‘Better project result’, ‘Better working atmosphere’, ‘Better mutual understanding’) and the scope of the effects of the tensions (‘On the project’, ‘Beyond the project’, or both).

The activity that most frequently led to positive experiences was the joint development of targets (74%, *n* = 23). Furthermore, the majority of tensions referred to in the context of this questions led to better project outcomes (41%, *n* = 15), with ‘Better working atmosphere’ (30%, *n* = 11) and ‘Better mutual understanding’ (30%, *n* = 11) being as frequently mentioned. Two participant statements describe this effect as follows:

*What was positive is the shared desire to finish the project on a positive note, and to see what everyone is contributing to achieve that goal. It was helpful to point out what this means to each partner and to create a shared understanding, including how the tension came up. With the shared understanding, a common solution can be found.*

*In the project it is therefore important to repeatedly make these areas of tension visible through communication and to bring about a change of perspective from time to time among the individual actors. This requires a high degree of communication skills and is essential for project management. Another experience is that conflicts and positive conflict resolution have a very positive effect on the project. It allows to develop understanding and trust, and to foster a very good working atmosphere. Therefore, the learning effect for me is not to shy away from conflicts, (but) to accept them as something positive to create trust and to gain a good "flow" for the project.*

A much clearer pattern emerged regarding the impact of tensions on the course of a project. According to 78% of participants (*n* = 25), the effects were limited to the project, 15% (*n* = 5) saw effects on the project and beyond, and only 6% (*n* = 2) saw effects exclusively beyond the project.

*A project meeting that addressed the tensions and conflicts. Different points of view could be communicated on a factual level. This made further cooperation much easier. This was very valuable for the further course of the project.*

*Because of the conflicts, there were more meetings so that everyone could voice their concerns. It was thus possible to understand the opinions of the others a little better and to incorporate this experience into future projects, so that they would be prepared for the occurrence of such conflicts.*

## Discussion

The results show that tensions and dealing with them have an impact on the course of IF projects, occasionally even beyond the project collaboration itself. The complexity of the implementation of integrated care projects, in terms of content, and the framework conditions of the IF are both major factors in the emergence of tensions. This becomes clear through the occurrence of tensions in the process phase of project implementation most frequently mentioned in the survey – both in terms of content and administration – and firmly places this phase in the centre of the analysis and of developing intervention approaches to deal with these tensions constructively and pre-emptively.

It has also been shown that tensions resulting from the intentional integration of different actors in the interorganisational, intersectoral, interprofessional, and interdisciplinary project consortia of the IF cannot always be prevented, e.g., through better planning. Conflicts in cross-sectoral collaboration in IF projects thus exhibit consistency and must be anticipated and considered when implementing projects. This assessment should not be viewed in an exclusively negative manner, since the dynamic consideration of contradictions and tensions can have both negative (e.g., delays and deviation from project objectives) and positive effects (e.g., improved communication and better understanding for others) on the course of the project, if it is done reflexively [22, 23, 40]. For example, the survey showed that a more productive work atmosphere, better understanding between the actors involved in the project and, above all, better project outcomes can be achieved by dealing with tensions. Projects in which the interests of the various consortium partners are successfully reflected and moderated can therefore have better results than those in which individual partners do not see their interests considered.

### Tensions as basis for consensual cooperation and decisions

To achieve a more successful cooperation between consortium partners in the context of integrated care projects requires constructive interaction by adapting existing procedures and working methods. It is necessary to support and enable the different stakeholder groups to transform their cooperation structures into new forms of collaboration as required by the project consortium or by the delivery of integrated care [15, 16]. This concerns all the stakeholders involved in a consortium or project rather than any individual groups. A coordinated (and facilitated) process of open discussions, in which the differences underpinning conflicts can be aired and discussed, would pave the way for consensual decisions to be made that consider the different perspectives, and ultimately increase the potential for better quality and successful project outcomes, which is also reflected in the survey results.

This approach also chimes with findings from group research, which prove that heterogeneous groups have an advantage over homogeneous groups. Indeed, it has been shown that in very homogeneous teams, with a strong sense of belonging, group cohesion can develop and, when combined with dysfunctional decision-making processes, such groups are less likely to produce successful outcomes [43].

Conflicts can thus function as the initial spark which breaks through (potential) existing cooperation barriers that (might) have emerged as a result of too much harmony, perspectives remaining unchallenged, and actors being stuck in their own position. In an open discussion, which allows differences to be leveraged rather than suppressed, it is possible for new trajectories and development perspectives to emerge. Furthermore, the stakeholders involved can express their own views, expectations and objectives, making these more transparent. On the basis of mutual understanding, individual positions can be clarified, and any rigid demands imposed by partners be adjusted. This opens the opportunity for a consensus or at least a compromise in which the different perspectives are considered based on the lowest common denominator. Activities that can support these processes include thoroughly searching for alternatives, a robust examination of the facts, and the consideration of risks (and opportunities) offered by the partners in the project team [43].

### Necessary conditions

To enable and foster this process, conditions must be adapted at micro and macro level.

Our survey results show that the tensions in IF projects predominantly fall into the categories *Performing, Belonging*, or *Performing::Belonging* (cf. Fig. 6). These tensions are primarily rooted in conflicting beliefs, values, and objectives, are project- and process-specific, and formulated at pivotal junctures in the collaboration – which is indicative of conflicts of interest. It is therefore essential that strategies enable actors to become aware of and reflect upon their own and each other's work methods and ideas about collaboration [22, 23]. At the micro level, the building of understanding and consensus must therefore be seen as a continuous process that uses the tensions arising in the course of an innovation's evolution to drive the project forward. The stakeholders involved must accept that ongoing dialogue and a culture of discussion and understanding of conflicts are central components and key drivers in cross-sector collaboration. In this context, another essential aspect that has emerged from the survey has to be considered. Participants emphasized the need to facilitate the development of a tangible and contractually fixed goal shared by all consortium partners. Conversely, pursuing shared aims and objectives in turn reduces tensions and controversies, as transparency regarding the intended outcome serves as a key point of orientation in debates and can thereby reduce uncertainties.

Furthermore, the results of the survey highlight the central importance of dealing with tensions as part of a dynamic approach to project management. Acting as a neutral authority, a central project coordinator could manage the processes, integrate the different perspectives within the consortium longitudinally, and deal with any emerging tensions as they arise. At the meso level, resources and structures should therefore be made available to establish project management as a standard component of IF projects, and in this way promote not only a more constructive and value-enhancing approach to handling tensions but also improve the overall communication and interactions between actors. Other factors named in the survey, such as the character traits of the actors involved and the lack of openness and understanding of different perspectives and ways of working, can also be actively and more productively dealt with through these activities.

### Implications for project budgets and framework

Accordingly, what is needed are not only structural changes to be made to the funding instrument, but also corresponding time quotas to be incorporated into project planning to enable coordinated discussions and the development of mutual understanding among consortia partners. Such measures also require adequate funding. However, the IF's current framework conditions only permit the use of resources for this type of activity to a limited extent, which further increases the level of tension in the event of conflicts. Thus, when tensions surface it is not only the intensity of the debate regarding content, but also the restrictive specifications of the framework conditions that impact the actors and further impede constructive discussion and consensus-building [22]. Since the need for such coordination and moderation can vary over the course of a multi-year project, a flexible budget for longitudinal management tasks of continuous alignment and control that can be used on an ad hoc basis is a conceivable option to be able to better respond to these situations. The communication and coordination structures created in this way can be used not only to deal constructively with conflicts, but also for other activities that are crucial to the success of the project and the dialogue between partners.

### Limitations and opportunities for future research

Our study has limitations, which at the same time can offer avenues for future research. It is limited by the small sample size (*n* = 58) in particular regarding the feedback from health care providers (*n* = 7). Low response rates are, however, not unusual against the background of a web-based survey, which usually has lower rates than other survey formats, especially when surveying physicians [44]. Our response rate (83 out of 185 invitees, or 45%), then, rather testifies to the considerable interest in the topic. Future research could address this by setting up a larger study over a longer time period to increase the study population.

The study is also limited by the restriction to IF projects from Germany. A relevant starting point for future research could be the consideration of other (project) constellations and the transfer of the research question and analysis to other health systems. In addition, the study is subject to limitations related to the survey method used, such as differences in the interpretation of terms, the response to questions, nonresponse bias, and recall error. Another unknown is whether and how individuals who either did not respond or complete the survey perceive and deal with tensions. Thus, there may be individuals who experience tensions in their project environment but do not recognize them, or they may have developed a successful way of dealing with tensions without linking these approaches to the success of their project.

Hence, one future research option would be to fill these knowledge gaps by undertaking a qualitative analysis of selected projects (e.g., successful, unsuccessful, with and without known tensions). In addition, longitudinal research should also be conducted to complement the cross-sectional analysis of the present study to explore further project developments regarding the visibility of tensions, changes, and transformation processes that have occurred in the projects. This may not only provide insights into the successful implementation of innovation projects in integrated care, but also allow to predict tensions and be able to offer effective strategies.

## Conclusion

Developing a better understanding of tensions promises to strengthen integrated care initiatives and improve their successful implementation. Our study has explored the positive impact that tensions can have on the outcomes of integrated care IF projects, if properly managed to avoid their destructive effects. This would require not only changes to the external framework conditions, but also raising awareness of the subject within the projects. In particular, the following measures would be conducive to successful project implementation and outcomes: financial and administrative leeway in project implementation to adjust timelines, scope and responsibilities if needed, application of project management that is characterized by agility and thus enables flexible and anticipatory handling of emerging requirements, strengthening communication and coordination through clearly defined goals and creating an atmosphere of trust between the different partners, offering spaces for discussion in workshops and project meetings, and facilitating mediation. Those responsible for the framework and implementation of integrated care projects can learn from these valuable insights on the emergence of tensions, their impact, and how to deal with them constructively in IF projects.

Future research can complement this with qualitative analyses combined with longitudinal studies through which project developments are explored in terms of the visibility of tensions and transformation processes, ultimately offering insights into the successful implementation of innovation projects in integrated care and the ability to predict tensions and provide effective strategies.

### Supplementary Information

**Additional file 1. **Survey on cooperation in Innovation Fund projects.

## Data Availability

The datasets used and/or analyzed during the current study are available from the corresponding author on reasonable request.
