# Supplementary material for: Why should stakeholders consider the effect of tensions in collaborative innovation in healthcare—lessons learned from surveying integrated care projects in Germany
Source: BMC Health Serv Res. 2023 Nov 23;23:1292. doi: 10.1186/s12913-023-10323-y (PMC10668511; doi:10.1186/s12913-023-10323-y)
Supplement: Supplementary file 1 — Additional file 1. Survey on cooperation in Innovation Fund projects. [file 12913_2023_10323_MOESM1_ESM.docx]

**Survey on cooperation in Innovation Fund projects**

| **Question 1** |
| --- |
| What fundamental differences occurred during the course of the Innovation Fund project? (Multiple answers possible) |
| 1. Different objectives of the consortium partners [elaborate explanation in comment field] 2. Different time horizon of the consortium partners [elaborate explanation in the comment field] 3. Different working methods of the consortium partners [elaborate explanation in comment field] 4. Different ideas about the cooperation of the consortium partners [elaborate explanation in comment field] 5. Other [please elaborate in the comment field] |
| [*Comment field* to elaborate on the answers] |

| **Question 2** |
| --- |
| Which personal circumstances reinforce the fundamental differences between the consortium partners? (Multiple answers possible) |
| 1. Lack of transparency and mutual trust between the consortium partners. 2. Lack of understanding for the opinions and characteristics of the other consortium partners 3. Existing cooperation experiences between the institutions of the consortium partners 4. Characteristics of the individual parties involved 5. Other [please elaborate in the comment field] |
| [*Comment field* to elaborate on the answers] |

| **Question 3** |
| --- |
| The combination of different institutional identities and logics in the consortium contains considerable potential for tension. |
| 1. Strongly agree; (2) Agree; (3) Neither agree nor disagree; (4) Disagree; (5) Strongly disagree |
| [*Comment field* to elaborate on the answers] |

| **Question 4** |
| --- |
| Which structural factors reinforce fundamental differences between the consortium partners? (Multiple answers possible) |
| 1. General framework and funding structure (e.g., no room for budgetary or content adjustments, tendering procedure) [please specify in comment field] 2. Bureaucratic requirements (e.g., reporting and documentation requirements) [please specify in comment field] 3. Contractual arrangements for project execution (e.g., performance-based dependency, lack of binding force) [Please specify explanation in comment field] 4. Complexity of project execution in terms of content (e.g., difficulties in recruitment or influence of other actors) [Please specify explanation in the comment field] 5. Other [please elaborate in the comment field] |
| [*Comment field* to elaborate on the answers] |

| **Question 5** |
| --- |
| The structural frameworks of Innovation Fund projects contain considerable potential for tension. |
| (1) Strongly agree; (2) Agree; (3) Neither agree nor disagree; (4) Disagree; (5) Strongly disagree |

| **Question 6** |
| --- |
| In which phase of the Innovation Fund project did fundamental differences between the consortium partners (individuals/interest groups) become apparent? (Multiple answers possible) |
| 1. Problem Identification Phase: In analyzing the underlying supply shortage, the structural or process issue 2. Solution Finding Phase: In the development of an idea or strategy or a new form of service to eliminate the deficit 3. Project Development and Application Phase: In the concretization of the conceptual approach as an Innovation Fund project 4. Project Implementation Phase (in terms of content): During the implementation, recruitment and realization of the work packages incl. adjustment and follow-up due to unforeseen developments and events 5. Project Implementation Phase (administrative): During the implementation of administrative requirements such as reporting and communication as well as operational project management 6. Project Completion Phase: In the assessment of the effectiveness, the evaluation of the project and the preparation of the report 7. Project Transfer Phase: Dissemination and consolidation of the project's findings |

| **Question 7** |
| --- |
| How did the fundamental differences affect the course of the Innovation Fund project? (Multiple answers possible) |
| 1. Strongly divergent views regarding structures and processes in the project (e.g., control vs. flexibility, collaboration vs. competition) 2. Strongly divergent views regarding objectives in the project (e.g., efficiency vs. quality, stability vs. dynamics) 3. Strongly divergent views regarding the willingness to change established procedures and working methods (e.g., existing processes in collaboration vs. new processes in the project) 4. Strongly divergent views regarding the interests to be pursued (e.g., competing values, competing understanding of roles) 5. Other [please elaborate in the comment field] |
| [*Comment field* to elaborate on the answers] |

| **Question 8** |
| --- |
| What are the institutional affiliations of the consortium partners (individuals/stakeholders) where these impacts have become apparent? (Multiple answers possible) |
| 1. Health insurance 2. Health care provider 3. Association of Statutory Health Insurance Physicians/Professional Association 4. University/scientific institute 5. Manufacturer of a digital application/platform 6. Other [please elaborate in the comment field] |
| [*Comment field* to elaborate on the answers] |

| **Question 9** |
| --- |
| The effects of fundamental differences are usually predictable, and resulting conflicts can be avoided by planning well in advance. |
| (1) Strongly agree; (2) Agree; (3) Neither agree nor disagree; (4) Disagree; (5) Strongly disagree |
| [*Comment field* to elaborate on the answers] |

| **Question 10** |
| --- |
| The effects of fundamental differences cannot be avoided in general and require a dynamic approach to the tensions or conflicts that arise as a result. |
| (1) Strongly agree; (2) Agree; (3) Neither agree nor disagree; (4) Disagree; (5) Strongly disagree |
| [*Comment field* to elaborate on the answers] |

| **Question 11** |
| --- |
| The occurrence of tensions and conflicts as a result of fundamental differences influences the progress of Innovation Fund projects. |
| 1. Strongly agree; (2) Agree; (3) Neither agree nor disagree; (4) Disagree; (5) Strongly disagree |
| [*Comment field* to elaborate on the answers] |

| **Question 12** |
| --- |
| The occurrence of tensions and conflicts as a result of fundamental differences threatens the success of a project and poses an existential threat to it. |
| 1. Strongly agree; (2) Agree; (3) Neither agree nor disagree; (4) Disagree; (5) Strongly disagree |
| [*Comment field* to elaborate on the answers] |

| **Question 13** |
| --- |
| Which approach to managing fundamental differences in the project helps to resolve any resulting conflicts? (Multiple answers possible) |
| 1. Activities to develop an understanding of the consortium partners (e.g., their goals, interests, and ways of working) 2. Activities to develop a mutual objective in the project (e.g., common milestones, discussion of requirements, deliverables, and procedures) 3. Activities to reduce the requirements associated with the funding structure and processes (e.g., facilitating and making financial and content adjustments more flexible) 4. Activities to provide facilitation of consortium partners and their actions (e.g., regular meetings for project information, coordination, and transparency) 5. Other [please provide explanation in the comment field] |
| [*Comment field* to elaborate on the answers] |

| **Question 14** |
| --- |
| Dealing with tensions and conflicts caused by fundamental differences has a negative impact on the course of the project (e.g., delays, deterioration of results, deterioration of the general atmosphere in the project). |
| 1. Strongly agree; (2) Agree; (3) Neither agree nor disagree; (4) Disagree; (5) Strongly disagree |
| [*Comment field* to elaborate on the answers] |

| **Question 15** |
| --- |
| Dealing with tensions and conflicts as a result of fundamental differences has negative effects extending beyond the course of the project (e.g., damage to trust between stakeholders). |
| 1. Strongly agree; (2) Agree; (3) Neither agree nor disagree; (4) Disagree; (5) Strongly disagree |
| [*Comment field* to elaborate on the answers] |

| **Question 16** |
| --- |
| Dealing with tensions and conflicts as a result of fundamental differences has positive effects on the course of the project (e.g. creating interfaces, accepting different points of view, increasing awareness). |
| (1) Strongly agree; (2) Agree; (3) Neither agree nor disagree; (4) Disagree; (5) Strongly disagree |
| [*Comment field* to elaborate on the answers] |

| **Question 17** |
| --- |
| Addressing tensions and conflicts resulting from fundamental differences has positive effects extending beyond the course of the project (e.g., creating a resilient level of discussion between different actors). |
| (1) Strongly agree; (2) Agree; (3) Neither agree nor disagree; (4) Disagree; (5) Strongly disagree |
| [*Comment field* to elaborate on the answers] |

| **Question 18 (optional)** |
| --- |
| What was your most negative experience with tensions and conflicts in the course of an Innovation Fund project and what was the cause of it from today's perspective? |
| [*Comment field* to elaborate on the answers] |

| **Question 19 (optional)** |
| --- |
| What was your most positive experience with tension and conflict in the course of an Innovation Fund project, and what was so positive about it? |
| [*Comment field* to elaborate on the answers] |

| **Question 20** |
| --- |
| What is your institutional affiliation? |
| 1. Health insurance 2. Health care provider 3. Association of Statutory Health Insurance Physicians/Professional Association 4. University/scientific institute 5. Manufacturer of a digital application/platform 6. Other [please specify explanation in the comment field] |
| [*Comment field* to elaborate on the answers] |
